# Decomposing inequality of opportunity in child health in Tanzania: The role of access to water and sanitation

**DOI:** 10.1002/hec.4591

**Published:** 2022-08-23

**Authors:** Mkupete Jaah Mkupete, Dieter Von Fintel, Ronelle Burger

**Affiliations:** ^1^ University of Dar es Salaam Dar es Salaam Tanzania; ^2^ Stellenbosch University Stellenbosch South Africa

**Keywords:** child nutrition status, inequality of opportunity, maize prices, Tanzania, water and sanitation

## Abstract

Poor child nutrition is a major public health challenge in Tanzania. Large between and within regional nutritional inequalities exist in rural and urban areas. We looked at how locational circumstances hinder children from having an equal opportunity for good nutrition. We used the 2008/09 Living Standards Measurement Study data for Tanzania to identify the part played by water and sanitation in rural and urban inequality of opportunity in child nutrition. We used the dissimilarity index and the Shapley decomposition technique to quantify and decompose inequality of opportunity in nutrition. We find that 16% of the circumstance‐driven inequality of opportunity needs to be redistributed for equality of opportunity to prevail. We find that in rural areas, about 42% of the inequality of opportunity in nutrition is due to water and sanitation problems and 22% to child age. In urban areas, we find that the inequality of opportunity is related mainly to the child's sex, price fluctuations and intergenerational factors. The findings suggest that policies to improve water and sanitation coverage could help equalize opportunities for children in rural areas. In urban areas, policies that could help equalize opportunities require incentives to change social norms and behavior around feeding practices and vaccination.

## 1 INTRODUCTION

The 2020 Global Nutrition Report reveals that large inequalities in nutrition persist in low and middle income countries (Micha et al., 2020). Location is highlighted as one of the major factors that determines these inequalities. Stunting rates are higher for rural than urban children, and overall inequality in nutrition is due largely to mean differences between urban and rural areas. Yet, inequality within urban areas can be more pronounced than in rural areas despite urban areas having better average nutrition (Fotso, 2007; Menon et al., 2000; Van de Poel et al., 2007). Identifying the factors most responsible for the inequality in rural and urban areas has received insufficient attention in the literature despite its relevance for policy. This paper explores the pertinent factors that determine inequality of opportunity in nutrition among children in Tanzania and considers which factors are most amenable to being addressed by policymakers.

For policy to be effective in reducing inequalities, it must identify and address specific aspects of the problem. We focus on analyzing rural and urban differences in nutrition inequality. The characteristics we analyze therefore have an aggregate geographical dimension, over and above individual household circumstances and choices that determine why children have different health outcomes. Traditionally, these aspects have been framed in the context of the literature on inequality of opportunities. The literature sees inequality as having either “illegitimate” or “legitimate” sources, and policies as best targeted at resolving the former. Roemer (1998) defines illegitimate sources of inequality as social, genetic and biological factors over which individuals have no control and which result in inequality of opportunity, and legitimate inequalities as those that arise from behaviors and decisions (such as for instance smoking, for which individuals can be held accountable), or “effort”. Roemer argues that to achieve a more just society, policy should equalize opportunities independently of circumstances. Fairness of outcomes, which was widely debated by social scientists and philosophers in the 1970–1980s, is a basic principle of the earlier literature on inequality of opportunity (Arneson, 1989, 1990; Cohen, 1989; Dworkin, 1981; Nozick & Williams, 1975; Rawls, 1971; Sen, 1979, 1985), and was formalized by Roemer (1993, 1998, 2002). This principle envisions equal opportunity across the same levels of effort and across different contexts with unequal initial circumstances. That is, economic systems should be designed to compensate for circumstance‐driven inequality (the things people can't help) and reward effort‐driven inequality (the things they can help).

However, this framework may not be fully appropriate for analyzing child stunting and thus requires adapting. Children under 5 years fall well below the age of consent threshold of 18 years (Brunori, 2017; Carrieri & Jones, 2018), beyond which people are considered to be responsible for their own actions. By this definition, all inequalities below the age of 18 could be classified as illegitimate. Further, inequality in child nutrition arises from circumstances beyond the control of households in which children live (the traditional “circumstances”) and from the choices of the parents rather than those of the children. From the child's perspective these can be viewed as circumstances, or the intergenerational transmission of inequality through parental effort. This makes it impossible to respect the compensation principle ‐ the idea that there should be redistribution that compensates children for the inequality resulting from their parents' choices (Jusot et al., 2013).

Even if all sources of inequality in nutrition are illegitimate, that is, circumstantial, from children's perspective, it is nevertheless desirable to prioritize factors that are most amenable to policy interventions. We therefore propose an alternative classification that also takes geographical dimensions into account and therefore aids in thinking about solving urban‐rural inequalities. The policymaking environment – including the public investment in and service delivery for commonly known as water, sanitation and hygiene (WASH) infrastructure and food market conditions – affects all households' ability to ensure that their children are adequately nourished. Water and sewerage infrastructure and services are an important determinant of geographical differences in nutrition outcomes. Under‐served by such infrastructure and services, we would expect rural areas to exhibit poorer nutritional outcomes. Policymakers need to create incentives for parental effort and leverage resources for improving service delivery in remote areas. The former requires co‐operation between policymakers and households, the latter more narrowly the commitment of policymakers.

Given this policy‐sensitive classification, we hypothesize that geographical nutritional inequalities can be addressed by tackling rural‐urban inequalities in infrastructure investments over the long run. Inequality of opportunity in child nutrition in low and middle income countries is often driven by geographical inequalities in service delivery. Geographical inequalities in access to safe WASH are evident in regional, national, subnational and rural and urban areas, with large disparities observed in sub‐Saharan Africa (WHO/UNICEF, 2015; Deshpande et al., 2020). Inadequate WASH has been associated with poor child growth manifested in bouts of diarrhea (Fink et al., 2011; Kumar & Vollmer, 2013), infectious diseases (Russell & Azzopardi, 2019; Waruiru et al., 2004) and environmental enteric dysfunction (Humphrey, 2009; Lin et al., 2013; Mbuya & Humphrey, 2016). Our paper focuses on the contribution of WASH to inequality of opportunity in child nutrition in rural areas.

Child health inequality is widely discussed in the literature that connects socioeconomic health inequality with socioeconomic inequality (Hong & Mishra, 2006; Kamal, 2011; Pathak & Singh, 2011; Subramanyam et al., 2010; Van de Poel et al., 2008; Van de Poel & Speybroeck, 2009; Wagstaff et al., 1991; Wagstaff & Watanabe, 1999; Zere & McIntyre, 2003). However, this literature treats inequality due to factors such as water and sanitation as a secondary problem. Income inequality is considered to be the primary source of health inequality, while inequality in WASH is only a symptom of income inequality (Aizawa, 2019). The inequality of opportunities literature, on the other hand, deals broadly with circumstances hindering equal access to WASH among children (Jemmali & Amara, 2018; Saidi & Hamdaoui, 2017), but pays little attention to understanding how circumstances of inadequate WASH contribute to the inequality of opportunity in health. Similarly, while recognizing the spatial dimension of inequality of opportunity, the inequality of opportunities studies do not distinguish which circumstances matter for the large geographic component of health inequality. Some circumstances are likely to matter more in rural areas and less in urban areas because of factors such as differences in the distribution of circumstances, culture and norms, level of education, and nature of economic activities.

Our study estimates the inequality of opportunity in child nutrition in Tanzania and assesses the contribution of access to water and sanitation to health inequality against the contribution of intergenerational factors and household choices. It helps explain how water and sanitation coverage and distribution affects inequality of opportunity in childhood. It also contributes to the inequality of opportunities literature by decomposing the inequality of opportunity by location to establish which of the policy‐sensitive circumstances matter more in rural and urban areas.

## 2 METHODS

### 2.1 Data and variables

This study uses the 2008/09 World Bank Living Standards Measurement Study‐Integrated Surveys on Agriculture (LSMS‐ISA) data for Tanzania. We chose this dataset for its rich set of variables. In particular, it allowed us explore the contribution of two early life factors, breastfeeding and vaccination, to the inequality of opportunity. These two variables were collected only in the first round of the LSMS‐ISA panel survey so we restricted the analysis to the 2008/09 data. Our results are therefore representative of a time when stunting was more common in Tanzania than it is today and access to water and sanitation less common. The survey collected a wide range of information on household characteristics, food and non‐food expenditure, children's parents, anthropometric information, child‐specific characteristics and community variables such as hospital availability. The survey covered 3265 households.

#### 2.1.1 Measuring child nutrition

The outcome variable for this study is binary, indicating whether a child is stunted or not. According to the World Health Organization (WHO, 2006), a child with a height‐for‐age *Z*‐score (HAZ) 2 standard deviations below the benchmark is defined as being stunted and this condition is indicative of chronic malnutrition (Karra et al., 2017; Perumal et al., 2018). The focus on stunting was motivated by the high prevalence rate in Tanzania at the time and its strong correlation with future opportunities. Unlike acute malnutrition, where the consequences can be reversed through timely treatments, the consequences of chronic malnutrition tend to persist over time (Andersen et al., 2017). We select stunting as a primary opportunity variable indicating the overall access to adequate nutrition for children in Tanzania.

#### 2.1.2 Circumstance variables

We consider two sets of circumstance variables. The first represents a set of factors over which neither children nor the previous generation have control. These are contextual factors that can be categorized as macroeconomic (access to water and sanitation infrastructure and maize prices) and biological (child age and sex) variables.^1^ Inadequate water and sanitation exposes children to infectious diseases and parasites that prevent the body's absorption of nutrients (Andersen et al., 2017; Mshida et al., 2018). Following previous studies (Van de Poel et al., 2007; Victora et al., 2005), we consider a household as having access to clean water if the primary source of water is either piped water, bottled water, a protected dug well or spring water, a cart with a tank or a drum, or a tanker‐truck. We defined access to sanitation as adequate if the main toilet facility used by a household is a pit latrine with a washable slab, VIP (ventilated improved pit) latrine, flush or ecological sanitation toilet. We hypothesize that unequal local availability of affordable and nutritious food is a determinant of cross‐community nutrition inequalities. Food prices stand proxy for the quality of diets and quantity of food available to children in the local economy, and are an important determinant of child malnutrition (Brenton & Nyawo, 2021; Grace et al., 2014; Lee et al., 2016; Woldemichael et al., 2017). We use regional maize prices that were derived from household food expenditures and quantities bought, as reported by the survey respondents. Research shows that the growth paths of children from high‐ and low‐income families begin to diverge once breastfeeding stops and children depend on food from other sources. Nutrition inequality arises because access to affordable food sources is unequal. From the age of 6 months, the gap in child growth between high‐ and low‐income families tends to increase and then starts to stabilize at 30 months (Andersen et al., 2017). The sex of a child is also regularly found to be a determinant of child growth in developing countries where cultural preferences for a boy or a girl are predominant.

The second set of variables relates to the effort of the previous generation but we classify them as circumstance variables because children have no control over these factors. Mother's education, age at birth of child, breastfeeding, vaccination, household consumption and household size all fall in this category. These circumstances, to a large extent, are due to the parents' behavior or own effort. For example, some parents may be less inclined to breastfeed and vaccinate their children because of cultural beliefs and perceptions, leading to different children's health outcomes. Breastfeeding improves children's immunity against infectious diseases and reduces the risk of various forms of malnutrition (Scherbaum & Srour, 2016; Stuebe, 2009). The WHO (2013) recommends that a child should be exclusively breastfed for 6 months, and breastfeeding should continue until 2 years of age; however, most children in Tanzania are not exclusively breastfed (Dede & Bras, 2020; Shirima et al., 2001; Victor et al., 2013). Studies also found overlap and interaction between malnutrition and infectious diseases and thus vaccination is particularly important for malnourished children (Prendergast, 2015; Savy et al., 2009). The relevance of household income and household size for child growth and development has been widely documented in several studies (Agee, 2010; Costa et al., 2018; Horton, 1986; Iftikhar et al., 2017; Kirk et al., 2018).

It is safe to make a normative assumption that the importance of these circumstances differs in rural and urban areas. For example, since the urban population enjoys high coverage of basic services (Jiménez Fernández de Palencia & Pérez‐Foguet, 2011; Mwakitalima et al., 2018), differences in water and sanitation are more likely to matter for health inequality in rural than urban areas. Because of high rates of early marriage and pregnancy, we do not expect teenage pregnancy to have a large influence on inequality (Kassa et al., 2018). Although parents in rural areas have lower average education levels than those in urban areas, higher inequality in parent's education in urban areas is more likely to imply higher inequality in children's health in urban than in rural areas. While low vaccination rates seem to be concentrated in rural areas, Moshi et al. (2021) found that exclusive breastfeeding is hardly practised in either area. We hypothesise that vaccination's influence depends largely on the distribution and interaction with other households' dietary‐related factors.

### 2.2 Estimation strategy

#### 2.2.1 Dissimilarity index and Human Opportunity Index

We draw on an established methodological framework to analyze child health inequalities (Barros et al., 2008; Paes de Barros et al., 2009; World Bank, 2006; Yalonetzky, 2012). We first compute a dissimilarity index (*D*‐index) to quantify the inequality of opportunity for each circumstance variable. We then calculate the Human Opportunity Index (HOI) to assess the coverage of these opportunities. Following Paes de Barros et al. (2009), the *D*‐index measures the weighted absolute differences in groups' mean access to different circumstances relative to the population mean. It describes the extent of misallocated opportunities that would have to be redistributed to ensure equitable access to these opportunities.

We estimate the D‐index in three steps. In the first step, we estimate the probabilities of each child accessing opportunities conditional on *m* circumstances (*x*_1_, *x*_2_, *x*_3_, …, *x*_*m*_) using a logit model.

(1) $$\ln\left(\frac{\mathrm{Pr}\left(\;I=1|x_{1},x_{2},\text{…}.,x_{m}\right)}{1-\mathrm{Pr}\left(I=1|x_{1},x_{2},\text{…}.,x_{m}\right)\;}\right)=\sum_{k=1}^{m}h_{k}\left(x_{k}\right)$$

In the second step, we use the coefficients obtained in the first step to predict the probability of access to opportunity for each child in the sample.

(2) $$\hat{p}=\frac{\exp\left(\hat{\beta_{0}+\sum_{k=1}^{m}X_{ki}\hat{\beta_{k}}}\right)}{1+\exp\left(\hat{\beta_{0}+\sum_{k=1}^{m}X_{ki}\hat{\beta_{k}}}\right)}$$

In the final step, we estimate the probability of access to opportunity for the overall population and then use these as inputs to derive the D‐index.

(3) $$\bar{p}=\sum_{i=1}^{n}w_{i}\hat{p_{i}}$$

(4) $$D=\frac{1}{2\bar{p}}\sum_{i=1}^{n}w_{i}|\hat{p_{i}}-\bar{p}|$$

where $\hat{p_{i}}$ is the predicted probability of an outcome for unique circumstance *i*, $\bar{p}$ is the average probability for the population, and *w*_*i*_ is the sample weight. The *D*‐index ranges from 0 to 1 (0 − 100 in percentage). *D* = 0 implies equality of opportunity: a child's likelihood of accessing opportunities is not affected by macro or intergenerational circumstances. *D* = 1 implies extreme inequality.

The HOI measures the average coverage rate (prevalence) of opportunities discounted by how equitably the opportunities are distributed among the population (as measured by the *D*‐index). In other words, it represents the average coverage of opportunities that are distributed independently of a child's circumstances. The HOI is a product of the average access rate $\left(\bar{p}\right)$ and the *D* − *index*. The index is dependent only on coverage if there is complete equality (*D* = 0), and the index decreases as inequality increases (0 < *D* ≤ 1). Equation (5) implies that interventions can aim to increase coverage $\left(\bar{p}\right)$ or to reduce dissimilarity (*D*).

(5) $$HOI=\bar{p}\left(1-D\right)$$

#### 2.2.2 Shapley and oaxaca decompositions

We decompose the inequality of a child's nutritional opportunity into its sources using the Shapley decomposition (Shorrocks, 1982). This technique allows us to establish the marginal contribution of each circumstance to inequality of opportunity. Un‐like other decomposition methods widely used in the literature (e.g., the Wagstaff decomposition) the Shapley decomposition satisfies the path‐dependent property in that it is additively decomposable. This means that the marginal contribution of each circumstance and group adds up exactly to the total inequality (Davillas & Jones, 2020; Shorrocks, 2013). In addition to the Shapley decomposition, we also applied the Oaxaca‐Blinder decomposition (Blinder, 1973; Oaxaca, 1973) to the *D*‐index to establish factors that are specific to inequality of opportunity within rural and urban areas. The Oaxaca decomposition also calculates counterfactual estimates using own‐region coefficients and the distribution of the circumstances of the other region. That is, the counterfactual for rural areas is calculated using the coefficient of circumstances in rural areas and the distribution of circumstances in urban areas, and a similar approach is followed for urban areas. The counterfactuals help to tell us whether the observed rural‐urban difference in inequality of opportunity is driven by the difference in the distribution of the circumstances or the size of the effect of the circumstances on inequality in the two areas (Juárez & Soloaga, 2014).

## 3 RESULTS

### 3.1 Descriptive results

The summary statistics of the 2008/09 sample used in the analysis are given in the online appendix. A significant difference can be seen in the prevalence of stunting in rural and urban areas. On average more children in urban areas are exclusively breastfed than in rural areas. Significantly more children in urban areas have access to clean water and adequate sanitation than in rural areas. Household size in rural areas is significantly larger than in urban areas. The food price difference between the two regions is significant, with higher prices in urban areas. Urban households, as expected, have higher consumption than rural households.

**FIGURE 1:**
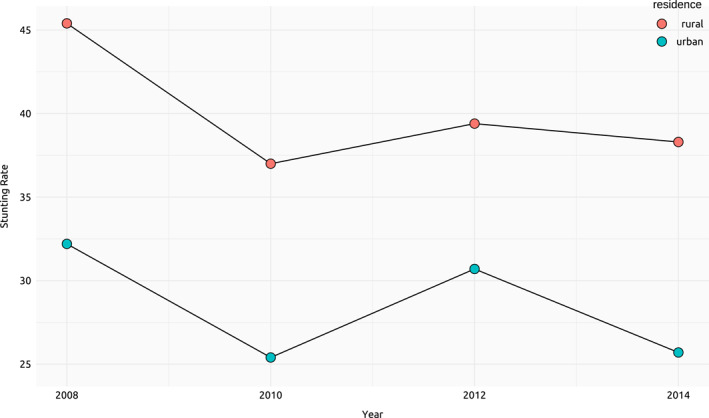
Trends in stunting rate, 2008–2014. *Data sources*: Living Standards Measurement Study‐Integrated Surveys on Agriculture (LSMS‐ISA)

Our main findings using the 2008/09 LSMS‐ISA data are representative of a time when child stunting was high. Figure 1 shows that the stunting rate remained extremely high between 2008 and 2014. More than a quarter of rural children were stunted in urban areas by 2014, while about 37% were stunted in rural areas. Even if stunting remained high, there were some improvements. One possible explanation for this trend is the expansion of water and sanitation services in Tanzania.

### 3.2 Trends in access to water and sanitation services

**FIGURE 2:**
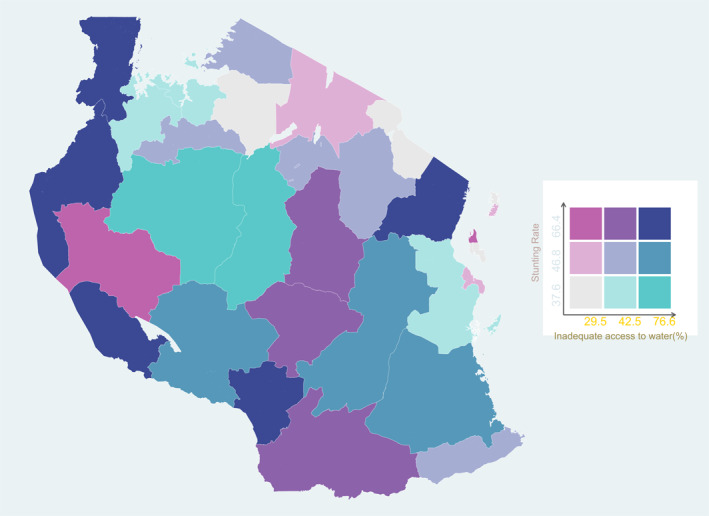
Access to Adequate Water Services and Stunting. *Data sources*: Living Standards Measurement Study‐Integrated Surveys on Agriculture (LSMS‐ISA) 2008

Figures 1 and 2 in the online appendix show trends in water and sanitation coverage in rural and urban areas using data from WHO‐UNICEF (2022). Fewer than 30% of rural households had access to basic water services in 2008 and fewer than 15% had access to sanitation services. Despite notable progress towards achieving the targets of clean water and sanitation listed under Sustainable Development Goal 6, by 2020 more than half of rural households did not have adequate water and nearly 75% had no improved sanitation services. In rural areas stunting has decreased since 2008, along with the improvements in water and sanitation. However, despite these improvements, access to services remained low up to 2020. These estimates suggest that inadequate water and sanitation continued to impose binding constraints on nutrition equality in rural areas up to 2020.

The situation is different in urban areas, where access to basic water services increased from a high base of 73% in 2008 to nearly 90% in 2020 (see Figure 1 in the online appendix. Significant proportions of urban households have received new sanitation services since 2008, but until 2020 fewer than 40% could access adequate sanitation.

### 3.3 Spatial relationship between WASH and stunting

**FIGURE 3:**
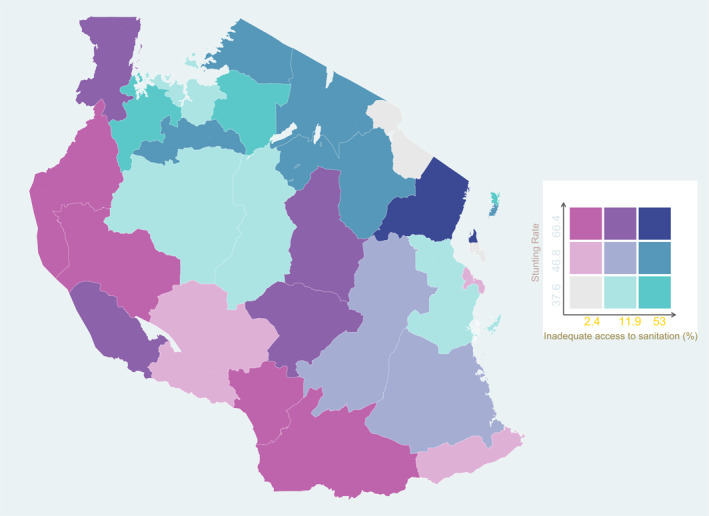
Access to Sanitation Services and Stunting. *Data sources*: Living Standards Measurement Study‐Integrated Surveys on Agriculture (LSMS‐ISA) 2008

Examining the relationship between WASH and stunting at a regional level, we find some corroboration of the hypothesis, but with exceptions. Exceptions are expected because of regional aggregation but also because WASH is not the only determinant of stunting. Figures 2 and 3 show the spatial relationships between stunting and inadequate access to water and sanitation respectively. High stunting rates and inadequate access to water occur together in the western, eastern and southwest regions. Selected regions in the North‐East have high access to clean water and low stunting rates. However, some districts in the center of the country have relatively low stunting rates despite having inadequate access to water. Figure 3 shows that for sanitation, the overlap between high stunting and inadequate access to sanitation is also strongest in the eastern and western regions.

Infrastructure provision is often not allocated according to need (TAWASANET, 2009). In many areas, funds are allocated to communities where access is already high, exacerbating inequalities. For example, in 2009, $5million more was disbursed to fund water projects in Arusha region than Tabora region despite the significantly low coverage rate in the latter (Hoffman, 2013). Similarly, TAWASANET (2009) examined water infrastructure funding and found that only 25% of district councils allocated a large share of finance to water projects in *marginalized* communities. As Figures 1 and 2 in the online appendix showed, authorities targeted water and sanitation infrastructure investments towards rural areas more broadly. However, as our maps show, coverage was uneven.

Be that as it may, the maps identify areas where expanding infrastructure could have potentially large effects on reducing stunting rates, while also addressing regional inequalities in stunting. Shifting the focus to specifically target regions with high stunting and low provision (such as the blue areas in Figures 1 and 2 in the online appendix could have meaningful effects on reducing stunting inequalities across regions and households.

The government's broader strategy has been to target rural areas. For example, the National Sanitation Campaign that was initially launched in 2012 by the Ministry of Health aimed at improving the rural households' access to adequate water and sanitation in all 26 regions (Mwakitalima et al., 2018). However, the maps show that the blanket approach has not reduced inequalities in access to these basic services between regions and thereby also not targeted regional inequalities in nutrition directly.

### 3.4 Correlates of stunting

Before delving into the analysis of inequality of opportunity in nutrition, we first present the correlates of stunting in the online appendix. Household consumption and access to clean and safe water are significantly related to a reduced likelihood of being stunted in rural areas. Maize prices are associated with an increased likelihood of stunting in urban children. Children of younger mothers are more likely to be stunted, but the association is significant only in urban areas. After the age of 5 months, the likelihood of children being stunted increases with age until 36 months and declines slightly thereafter in rural areas. In urban areas, children aged 6–24 months (the highly sensitive complementary feeding age) are 2.3 times more likely to be stunted than younger children aged 0–5 months.

### 3.5 Inequality of opportunity and decomposition

Table 1 shows that the value of the adapted *D*‐index is 0.16. A large part of the inequality of opportunity in child health is therefore attributable to circumstances. In other words, 16% of circumstance‐driven inequality would have to be redistributed from non‐stunted to stunted children to attain equality of opportunity. Given that it is unlikely that all the circumstances affecting child health are included in the analysis, the values of the *D*‐index presented should be regarded as lowerbound estimates. Nevertheless, this level of inequality of opportunity is similar to that reported in other contexts that also have a high prevalence of malnutrition (Hussien et al., 2016; Saidi & Hamdaoui, 2017).

**TABLE 1:** Decomposition of Inequality of opportunity in stunting (Oaxaca decomposition of D‐index

Distribution	Coefficients	
	Rural	Urban
Rural	0.035	0.046
Urban	0.030	0.046

Method	Absolute	
PdB (dissimilarity index)	0.088	
Ws (adapted DI)	0.157	

*Note*: The figures on the diagonal (in bold) are the own‐region PdB estimates, and the off‐diagonals are the counterfactuals. PdB stand for Paes de Barros et al. (2007) and WS for Wendelspiess and Soloaga (2013) indices. PdB is scale invariant but not translation invariant. WS is translation invariant but not scale invariant.

The top panel of Table 1 presents the results of the Oaxaca‐Blinder decomposition of inequality of opportunity in child health across rural and urban areas. The unadapted *D*‐indices for each region (rural and urban) are given by the values on the diagonal of the table. Off‐diagonals represent the counterfactual estimates. The inequality of opportunity estimates for rural and urban areas are 0.035 and 0.046, respectively. Stunting is therefore more unequally distributed across circumstances in urban than in rural areas. This result is aligned with the literature on socioeconomic inequality in malnutrition which finds higher inequality in urban than rural areas (Fotso, 2007; Menon et al., 2000; Van de Poel et al., 2007). The unadjusted *D*‐index is substantially lower in urban and rural areas than it is in the pooled sample (0.088). A large component of Tanzania's national inequality of opportunity is therefore determined by cross‐regional differences. However, the counterfactual estimates indicate that if children in urban areas were affected by circumstances similarly to rural children, their inequality of opportunity would come remarkably close to that of rural children. A similar result holds if we apply the urban counterfactual to rural children. It suggests that the levels of inequality would be the same in urban and rural areas if children faced the same circumstances.

Table 2 presents the results of the Shapley decomposition of the inequality of opportunity in nutrition. The results of the full sample indicate that water and sanitation are major contributors to inequality, explaining 36% of total inequality. Household consumption accounts for a further 24%, followed by child's age, which explains 15%. The child's sex appears far more important than intergenerational factors (represented by mother's education and mother's age at birth of child) in explaining inequality of opportunity. Child's sex contributes 9% of the variation and intergenerational variables contribute 2%.

**TABLE 2:** Shapley decomposition of inequality of opportunity in stunting

Variable	Full sample		Urban		Rural	
	Value	%	Value	%	Value	%
Maize price	0.005	5.27%	0.025	17.95%	0.003	4.73%
Consumption	0.021	23.53%	0.016	11.67%	0.011	14.29%
Breastfeeding	0.005	5.74%	0.017	12.43%	0.002	3.33%
Vaccination	0.000	0.21%	0.008	5.89%	0.001	1.94%
Mother's educ. and age at child's birth	0.002	2.79%	0.016	11.55%	0.002	3.32%
Water and sanitation	0.032	36.36%	0.000	−0.33%	0.031	41.50%
Healthcare	0.000	0.16%	0.001	0.69%	0.001	0.70%
Household size	0.002	2.19%	0.007	4.75%	0.002	2.80%
Child's age	0.013	15.09%	0.006	4.25%	0.016	22.36%
Child's sex	0.008	8.65%	0.041	29.97%	0.004	5.05%
TOTAL	0.088	100.00%	0.137	100.00%	0.074	100.00%

The disaggregated analysis by region type in columns (5) and (7), however, shows that the regional distinction is important. Access to clean water and improved sanitation contributes 42% of the inequality of opportunity in nutrition in rural areas, while the contribution is negligible in urban areas. A further 22% of the inequality of opportunity in nutrition in rural areas is related to the child's sex and another 14% to household consumption. The rest of the circumstances contribute a relatively small amount ranging from 2% to 5%. Un‐like the case in rural areas, child's age and maize prices are the leading contributors in urban areas, contributing about 30% and 18%, respectively. Intergenerational factors, such as breastfeeding and a household's socioeconomic status, each contribute 12% of the total inequality in the urban setting.

Table 3 shows that the opportunity for access to clean water is limited to only 56.8% of children in rural areas, with 2.9% of the opportunities needing to be reallocated to attain equality of opportunity. Similarly, for urban areas, 83.5% of children have access to clean water, with only 3% of the opportunities needing to be reallocated to attain equality of opportunity. The results also show that despite the magnitudes of the dissimilarity indices being very similar across urban and rural areas, there is higher inequality of opportunity across region types emanating from unequal coverage of water services. Furthermore, 86% of children in rural and 99% in urban areas live in a house with improved sanitation. The overall dissimilarity indices in both regions are small. Access to healthcare services is limited to slightly above 50% of children in both regions, and the *D*‐indices are relatively high at around 5.

**TABLE 3:** Inequality of opportunity in access to basic services in rural and urban areas

	Water			Sanitation			Healthcare		
	C	D	HOI	C	D	HOI	C	D	HOI
Rural	56.8	2.9	55.2	86.0	1.8	84.4	54.9	5.5	51.9
Urban	83.5	3.0	81.0	99.0	0.5	98.6	53.3	4.9	50.7

*Note*: Coverage (C), Dissimilarity (D) and Human Opportunity Index (HOI).

## 4 DISCUSSION

The analysis demonstrated that location plays an important role in shaping the inequality of opportunity in nutrition. Stunting is more unequally distributed in urban than in rural areas, confirming the findings of studies on socioeconomic inequality in child health (Fotso, 2007; Menon et al., 2000; Van de Poel et al., 2007). The decomposition further indicates that the circumstances that influence inequality in child nutrition are different in rural and urban areas. Water, sanitation and child age represent 66% of the total inequality in rural areas, of which 42% of the inequality results from water and sanitation on its own. Our finding that water and sanitation is a dominant contributing factor to the inequality of opportunity in nutrition in rural areas is aligned with the existing evidence of a strong link between WASH and child growth found in the literature (Kwami et al., 2019; Rah et al., 2015; Smith & Haddad, 2015). This finding can be explained by a significant gap in the coverage of clean water that disadvantages rural areas, as revealed by our Human Opportunity Index (HOI) analysis.

The largest part of inequality of opportunity in nutrition in Tanzania's rural areas is therefore determined by factors beyond the control of children and their parents. These are factors that are most suitably addressed by public interventions such as improving access to clean water and sanitation in rural areas, where a high share still lack such access. Poor households in particular are likely to benefit from the universal provision of water and sanitation services, given the nature of the community‐level circumstances they face. Because water and sanitation services are commonly available to richer households, improving their availability might bring less improvement in child growth in urban areas.

Mobilising resources for universal water and sanitation coverage is perhaps only achievable over the very long run. Expanding these services is, furthermore, the prerogative of policymakers. Behavioral interventions that attempt to influence the effort levels and attitudes of parents, on the other hand, require the co‐operation of both policymakers and individuals from the older generations. Incentivising behavior change related to gender norms and roles is more complicated than extending the coverage of water provision. The progress made in Tanzania since the 2008 LSMS‐ISA survey suggests a concurrent decrease in stunting with the expansion of water and sanitation coverage, showing that this approach can have important benefits even in the short run, and aim to eliminate rural health inequality in the long run.

Increasing coverage without considering socioeconomic profiles may lead to unintended consequences. It is possible that interventions that reach richer households before they target poor households could exacerbate inequality of opportunity in child nutrition rather than reducing it. The time horizon for reducing inequality will also be later than planned as it takes time for poor households to be affected and to converge on better‐off households (Victora et al., 2000). Interventions that first target the poor—in the case of Tanzania that implies rural areas—have the most rapid effect on reducing inequality. Monteiro et al. (2009) found that approximately two‐thirds of the decline in stunting (from 37% to 7%) in Brazil between 1996 and 2007 was the result of the increased supply of water and sanitation services for people in the lowest wealth quintiles.

Success in improving child health and growth by increasing the coverage of water and sanitation has also been reported in India (Hammer & Spears, 2016), Argentina (Galiani et al., 2005), Peru (Checkley et al., 2004) and Mali (Harris et al., 2017). However, experience shows that investing in better water and sanitation has only a modest effect on improving child outcomes unless entire communities are targeted for universal coverage (WHO, 2014, 2019). Several household‐level intervention studies have reported little effect of improving WASH on reducing diarrhea and improving child growth (Humphrey et al., 2019; Luby et al., 2018; Null et al., 2018). In contrast, reducing open defecation at the community level has been shown to reduce stunting in India (Spears et al., 2013). In Tanzania, Gertler et al. (2015) showed that a child would gain a 0.44 standard deviation increase in growth if open defecation was eliminated in the entire village.

The situation is different in urban areas, where biological factors and parental effort play a larger role in determining inequality of opportunity in child nutrition. This means that investing in public infrastructure may not have the same impact as it would in rural areas. Rather, deeper behavioral changes are needed to change social patterns, requiring co‐operation between households, communities and policymakers. Unexpectedly, we find that the child's sex contributed to more than a quarter of the inequality of opportunity in nutrition in urban areas. Despite evidence that male children are more likely to be stunted than female children in sub‐Saharan Africa (Espo et al., 2002; Wamani et al., 2004, 2007; Zere & McIntyre, 2003), the effect of sex discrimination on child health outcomes is expected to be more prevalent in rural areas where child sex preferences persist. We were surprised to find that in urban areas, where parents have higher literacy rates and are exposed to new norms and cultural diversity, child sex preference contributed to child nutritional inequalities. The preference for boys is widely established in other developing countries such as Bangladesh, India, China and Pakistan; however, it is less pronounced in sub‐Saharan Africa (Filmer et al., 2008). Nonetheless, our findings are similar to those reported in Tunisia (Amara et al., 2017). They differ, however, from a study by Ersado and Aran (2014) in Egypt which found that girls were more likely to be stunted than boys. These findings are also in line with Hoyos and Narayan (2011), who showed that the sex of a child influences access to basic services opportunities in 47 countries, including Tanzania.

We found that intergenerational aspects such as mother's age at the child's birth and mother's education as well as early life child feeding practices (breastfeeding) were also far more important in urban than rural areas. Maternal years of education has been widely shown to be a strong predictor of child health (Schultz, 2002). While education interventions could be important in both rural and urban areas, reaching disadvantaged children in urban areas offers more potential for narrowing inequality of opportunity in nutrition. More educated mothers are likely to provide good care and ensure that their children use treatment and prevention services effectively (Caldwell, 1979, 1994). Education can also have spillover effects in equalising opportunities across factors such as breastfeeding and childbearing age, which are important determinants of child growth (Boyle et al., 2006). Nutritional education has proved effective in improving child health in Malawi and Pakistan (Khan et al., 2013; Ragasa et al., 2019).

Another interesting finding that emerged from our analysis was the large contribution of maize price fluctuations to the inequality of opportunity in nutrition in urban areas. About 18% of total inequality of opportunity could be attributed to this factor, far exceeding the contribution of household variables. These findings are drawn from the supplementary analysis provided in Table 3 in the online appendix. We found that maize prices negatively affected the growth of children who lived in households where there was no food production, mostly in urban areas. In contrast, the impact of prices was negligible for children from food‐producing households, mostly in rural areas. It has been shown elsewhere that higher food prices in urban than rural areas in most developing countries increases the vulnerability of urban children (Cohen & Garrett, 2010; Stage et al., 2010). These findings imply that policies that stabilise food prices or compensate households for price increases can help improve equality of opportunity in child nutrition particularly in poor urban contexts.

Household income or wealth was found to be the leading contributor to child health inequality in most studies on socioeconomic inequality in child health (Kien et al., 2016; Rizal and van Doorslaer, 2019) and inequality of opportunity in nutrition (Aizawa, 2019; Hussien et al., 2016) in other developing countries. In our study, however, we found that the contribution of household consumption, a close proxy for income, was relatively small compared to the contribution of basic water and sanitation services in both the full samples and the samples disaggregated by region type. This finding might explain the persistence of the high stunting rate and nutritional inequalities in Tanzania despite the significant decline in poverty levels in the past 25 years. Reducing poverty without considering its multidimensional aspects might not yield the intended outcomes, given the strong link between stunting and different aspects of poverty.

The strengths of our study lie in our conceptualisation of inequality of opportunity in child nutrition. While all factors are outside of the control of children, we show that a number of policy‐sensitive variables are especially important in addressing rural nutritional inequalities, albeit over the long run. However, because we had rich data on intergenerational circumstances, we were able to show that factors beyond the policy domain may hinder the short‐run reduction of inequalities in urban areas. Without well‐designed interventions to change social and behavioral norms, urban nutritional inequalities may persist despite infrastructural improvements like better water and sanitation coverage.

The main limitation of this study is that we used only one cross section from 2008/09. The LSMS‐ISA data for Tanzania is more than a decade old and many changes have occurred since then. Water and sanitation coverage has increased and stunting has decreased. While this would be aligned to our conclusion that investment in water and sanitation can reduce stunting and also inequality in child health, we have no firm evidence to support that line of causality. A dynamic analysis would be able to overcome this problem. However, the LSMS‐ISA surveys after 2008/09 do not permit this type of analysis, unless we ignore two important early life factors, breastfeeding and vaccination. We chose to use the 2008/09 LSMS‐ISA data because it includes those two factors. Combining the Demographic and Health Survey with LSMS‐ISA data could supplement the findings of our study. In addition, malnutrition in Tanzania demonstrates a spatial pattern. In our study, the lowest level for spatial disaggregation was rural and urban. Modeling malnutrition using spatial models such as geographically weighted regression models, which produce location‐specific determinants, could be relevant for targeting policy.

## 5 CONCLUSION

This study quantified the inequality of opportunity in nutrition among children under age five in Tanzania and assessed the circumstances that matter more in rural and urban areas. We used data from the LSMS‐ISA and applied the Shapley decomposition technique to the dissimilarity index (*D*‐Index) to gauge the contribution of each circumstance to the inequality of opportunity. We found that inequality of opportunity in child nutrition in Tanzania is attributable to a different set of circumstances in rural and urban areas. A large share of inequality of opportunity in rural areas emanates from access to water and sanitation followed by child age. Intergenerational factors—mother's education and her age at the birth of her child and early life child feeding practices—are more important in urban than in rural areas. The child's age and sex also have different effects in rural and urban areas. Child's age mattered more in rural areas and child's sex mattered more in urban areas. We also found that maize price fluctuations contributed a larger share of inequality of opportunity in nutrition in urban than in rural areas.

The findings highlight the areas of intervention that policymakers can intensify to reduce inequality of opportunity in child nutrition by targeting location‐specific factors. Increasing coverage of water and sanitation has the potential to reduce malnutrition and inequality of opportunity in nutrition in rural areas, albeit conditional on entire communities being reached. These factors can only be addressed on a large countrywide scale by policymakers and will probably only have an effect in the long run, once sufficient resources are mobilized to achieve these objectives. To increase the pace of reducing stunting, the government should prioritize the areas where high stunting and inadequate access to WASH services occur together, particularly in the western, eastern and southwest regions. Given the particular importance of intergenerational factors that relate to inequality in urban areas, behavioral change interventions to improve feeding practices and vaccination uptake and reduce teenage pregnancy could be used to address inequality of opportunity. However, because these interventions require changing behavior and habits, and often also social practices and norms, they are more complex to implement with success.

## CONFLICT OF INTEREST

The authors have no conflict of interest to declare.

## Supporting information

Supplementary Material S1

## Data Availability

The data that support the findings of this study are openly available in the World Bank central data catalog at https://microdata.worldbank.org/index.php/catalog/76.

## ENDNOTE

^1^
In some cases, inadequate water and sanitation may also be classified as an effort variable on the part of parents. This is because there is a strong link between income poverty and access to basic services. Parents may choose to relocate to areas with better water and sanitation provision when their incomes increase. Effort‐driven income inequality across families may lead to unequal access to water and sanitation services across children.
