# Supplementary material for: Decomposing inequality of opportunity in child health in Tanzania: The role of access to water and sanitation
Source: Health Econ. 2022 Aug 23;31(11):2465–80. doi: 10.1002/hec.4591 (PMC9805148; doi:10.1002/hec.4591)
Supplement: Supplementary file 1 — Supplementary Material S1 [file HEC-31-2465-s001.pdf]

Decomposing Inequality of Opportunity in Child Health in Tanzania: The Role of Access to Water and Sanitation

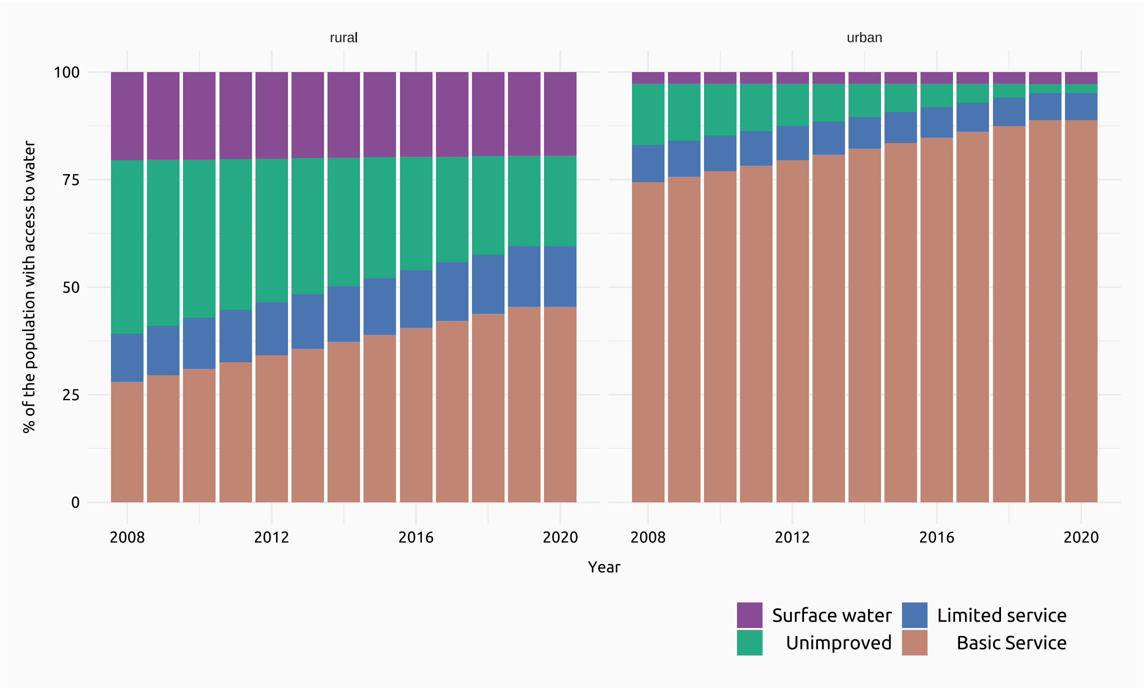

Figure 1: Coverage of Water Services in Rural and Urban Areas

Data sources: WHO/UNICEF Joint Monitoring Programme **?**

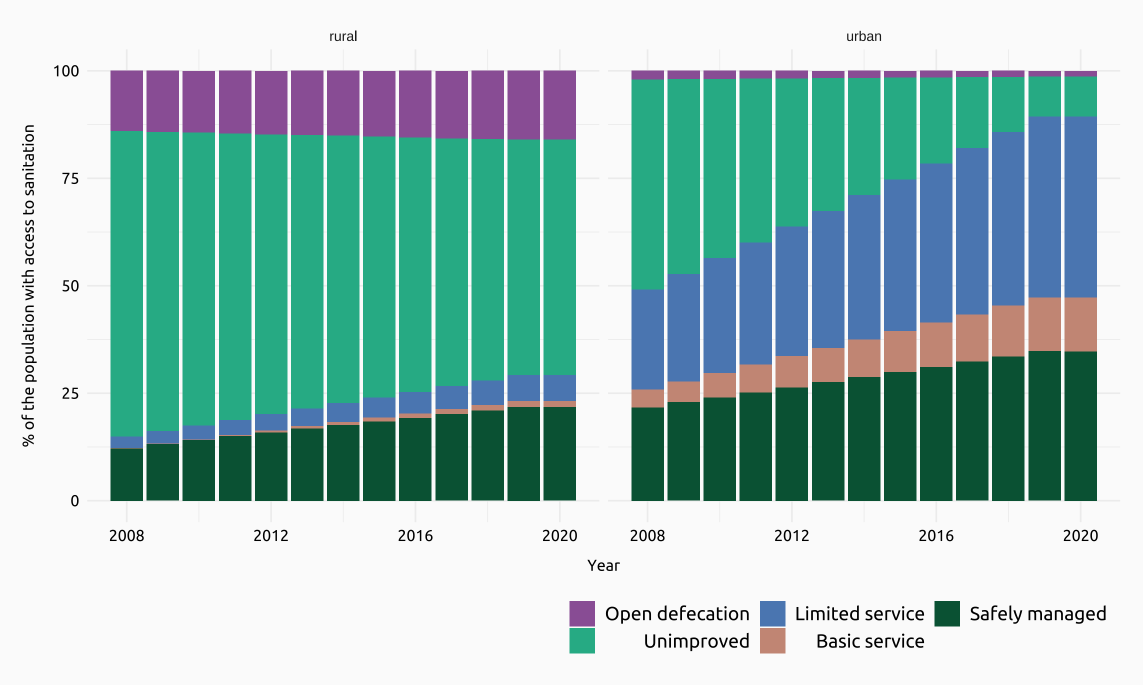

Figure 2: Coverage of Sanitation Services in Rural and Urban Areas

Data sources: WHO/UNICEF Joint Monitoring Programme **?**

Table 1: Summary statistics of household variables

Variable	Variable definition	Full Sample	Urban	Rural	Rural-Urban
Stunting	Height-for-age Z score <-2SD	0.42	0.322	0.454	0.132***
Maize price	Median price at district level in TZS in 2008	7.02	7.025	7.016	-0.009
Consumption (log)	Monthly household consumption in TZS	14.56	14.980	14.413	-0.567***
Breastfeeding	1 if a child was exclusively breastfed for six months	0.30	0.315	0.292	-0.023
Vaccination	1 if a child received a polio or measles vaccine	0.93	0.934	0.935	0.001
Age at birth	1 if mother's age at child's birth less than 18 years	0.02	0.017	0.020	0.003
Mother's education	Years of mother's schooling	5.657	7.443	5.039	-2.404***
Water	1 if a HH has access to piped water, bottled water, protected well, spring water, cart with tank or tanker-truck	0.64	0.847	0.569	-0.279***
Health care	1 if there is a hospital/ dispensary in the village	0.55	0.516	0.356	-0.160
Sanitation	1 if HH has access to improved sanitation: flush toilet, ventilated pit latrine, pit latrine with slab or ecological toilet	0.88	0.742	0.542	-0.200***
Household size	No. of people in HH	6.95	5.972	7.289	1.316***
Age (0-5)	Child aged between 0 and 5 mths	0.05	0.028	0.052	0.025**
Age (6-24)	Child aged between 6 and 24 mths	0.31	0.302	0.309	0.007
Age (25-36)	Child aged between 25 and 36 mths	0.22	0.215	0.216	0.001
Age (37-60)	Child aged between 37 and 60 mths	0.43	0.455	0.422	-0.033
Child=male	Child's sex: 1=male; 0=female	0.52	0.549	0.473	-0.076
N		2017	489	1528	

NOTES: Source: Own calculations from LSMS-ISA 2008/09

Table 2: Logit Regression results for stunting in rural and urban areas

	(1)<br>Full sample	(2)<br>Rural	(3)<br>Urban
log(Maize price)	0.641*	0.410	2.351**
	(0.344)	(0.359)	(1.037)
log(Consumption)	-0.277***	-0.251**	-0.183
	(0.081)	(0.102)	(0.179)
Breastfeeding	-0.152	-0.088	-0.254
	(0.105)	(0.121)	(0.244)
Vaccination	-0.142	-0.249	0.267
	(0.210)	(0.236)	(0.518)
Mother's age at child's birth	0.066	-0.241	1.270*
	(0.335)	(0.386)	(0.658)
Mother's education	-0.043	-0.029	-0.076
	(0.030)	(0.037)	(0.058)
Water	-0.304***	-0.316***	0.076
	(0.098)	(0.108)	(0.274)
Healthcare	0.067	0.072	0.078
	(0.095)	(0.109)	(0.203)
Sanitation	-0.506***	-0.402	-0.347
	(0.176)	(0.329)	(0.244)
HH size	0.015	0.009	0.022
	(0.013)	(0.015)	(0.038)
Age (0-5)	1.222***	1.106***	2.342**
	(0.266)	(0.285)	(1.091)
Age (6-24)	1.200***	1.205***	1.920*
	(0.272)	(0.293)	(1.094)
Age (25-36)	0.779***	0.784***	1.485
	(0.263)	(0.281)	(1.088)
Age (37-60)	-0.314***	-0.225**	-0.612***
	(0.093)	(0.107)	(0.196)
Constant	-0.974	0.346	-15.710**
	(2.617)	(2.893)	(7.223)
Observations	2017	1528	489

Own calculations from LSMS-ISA 2008/09. Robust standard errors in parentheses *p<0.10 **p<0.05 ***p<0.01.

Table 3: Shapley Decomposition of Inequality of Opportunity in Stunting

	Full sample		Maize producers		Maize non-producers	
Variable	Value	%	Value	%	Value	%
Maize price	0.003	4.44%	0.003	3.21%	0.035	22.67%
Consumption	0.010	13.80%	0.014	17.67%	0.001	0.76%
Breastfeeding	0.002	3.29%	0.005	5.85%	0.008	5.37%
Vaccination	0.001	1.75%	0.000	0.30%	0.004	2.65%
Mother's educ. & age at child's birth	0.006	8.57%	0.010	11.85%	0.038	24.64%
Water & sanitation	0.030	40.41%	0.029	35.81%	0.002	1.50%
Healthcare	0.001	0.69%	0.001	1.27%	0.005	3.07%
Household size	0.002	2.16%	0.003	3.13%	0.005	2.89%
Child's age	0.014	19.64%	0.011	13.58%	0.019	12.45%
Child's sex	0.004	5.24%	0.006	7.32%	0.037	23.94%
TOTAL	0.074	100%	0.081	100%	0.156	100%
